# Bridging the Divide between Manual Gating and Bioinformatics with the Bioconductor Package flowFlowJo

**DOI:** 10.1155/2009/809469

**Published:** 2009-10-07

**Authors:** John J. Gosink, Gary D. Means, William A. Rees, Cheng Su, Hugh A. Rand

**Affiliations:** ^1^Department of Computational Biology, Amgen, 1201 Amgen Court West, Seattle, WA 98119, USA; ^2^Department of Molecular Sciences, Amgen, 1201 Amgen Court West, Seattle, WA 98119, USA; ^3^Department of Biostatistics—Medical Sciences, Amgen, 1201 Amgen Court West, Seattle, WA 98119, USA

## Abstract

In flow cytometry, different cell types are usually selected or “gated” by a series of 1- or 2-dimensional geometric subsets of the measurements made on each cell. This is easily accomplished in commercial flow cytometry packages but it is difficult to work computationally with the results of this process. The ability to retrieve the results and work with both them and the raw data is critical; our experience points to the importance of bioinformatics tools that will allow us to examine gating robustness, combine manual and automated gating, and perform exploratory data analysis. To provide this capability, we have developed a Bioconductor package called flowFlowJo that can import gates defined by the commercial package FlowJo and work with them in a manner consistent with the other flow packages in Bioconductor. We present this package and illustrate some of the ways in which it can be used.

## 1. Introduction

Flow cytometry is a high-information content platform that is increasingly becoming a high-throughput platform as well [1]. Flow cytometers measure individual cells, and thus are capable of revealing subtleties of biology that other technologies cannot detect. Recent advances in instrumentation such as 4 and 5 color laser systems and the availability of reagents and protocols for assessing internal proteins and their phosphorylation state are serving to make flow cytometry a very important tool for understanding disease processes in human biology [2]. There is also a growing appreciation that it is important to assess cells not only in their quiescent state, but also in response to various stimuli [3]. This adds another layer of complexity to flow cytometry data sets. Powerful analysis tools are needed to properly explore and analyze data sets in which each sample has many stimuli, cell subpopulations, and phosphoprotein measurements.

 There are a number of challenges associated with the analysis of these large, complex flow cytometry data sets. The challenges can be divided into. (1) acquisition of high-quality data, (2) tools for data organization, annotation, and query, (3) tools for data manipulation, and (4) techniques and statistical methods for data analysis. All of these components are related and, done well, serve to reinforce each other. The first two of these tasks tend to be application- and lab-specific, while the latter two lend themselves well to the development of shared tools for all those faced with complex flow cytometry analyses. Similar to tools developed for microarrays, a set of packages is evolving in the Bioconductor community that holds great promise for flow cytometry data analysis. These packages which include flowCore [4, 5], flowQ, flowViz, flowUtil, flowStats, flowClust [6] and others all operate on a common set of core methods and classes for reading, transforming, gating and otherwise manipulating flow cytometry data.

 In the analysis of flow cytometry data it is important to be able to work with the gates that have been manually defined. Commonly these gates are defined in a commercial flow cytometry analysis package that is used, along with “cut-and-paste” and simple analysis packages such as Excel or Prism, to provide results. This becomes problematic when dealing with complex problems and large data sets. To address this problem, we have built a package that provides a way to extract data from one such commercial package, FlowJo (http://www.flowjo.com/), into the publicly accessible analysis platform R/Bioconductor. We chose to use FlowJo because it is amongst the most commonly used flow cytometry programs and it stores its session information in an open format. The package flowFlowJo can produce R data structures with either summary statistics or fully flowCore compliant objects representing the various gates, compensation matrices, and other related information embedded in FlowJo sessions. The goal of flowFlowJo is to make it easy, in R, to use compensation and gating information that has been produced using FlowJo. The flowFlowJo package provides the ability to work with both the raw data and the gating information in a powerful analysis environment that makes full use of the existing open source community efforts.

## 2. Software

### 2.1. Overview of the flowFlowJo Package

FlowJo is a commercially available software package used for the gating, visualization, and analysis of data from flow cytometry experiments. FlowJo saves its session information in an eXtensible Markup Language (XML) text file called a *workspace*. A workspace file contains all the information necessary to describe the gating structures, compensation, transformations, locations of the Flow Cytometry Standard (FCS) [7] files, graphs, and figures created by the user. FlowJo workspace files do not contain raw cytometry data.

The R package flowFlowJo is a set of methods and classes designed to extract the file locations, gates, compensation matrices, and some of the other information contained in FlowJo workspace files and return the information in a manner consistent for use with the Bioconductor flowCore packages. The flowFlowJo package can execute the following actions when supplied with the location of one or more FlowJo workspaces:

1. read and parse the workspace(s),
2. extract the location of all of the FCS files referenced in the workspace(s),
3. extract all of the intermediate and final gates as flowCore S4 class filters objects,
4. extract the spillover matrices,
5. extract the transformation settings,
6. organize the extracted information into a set of data structures so that all of the compensation and gating strategies described in the workspace(s) can be executed in R. In effect, this captures and executes much of the analysis workflow stored in the FlowJo workspace,
7. return a set of identically ordered lists containing all of the file locations, file names, filter objects, filter names, and compensation matrices.

These operations are typically done by an analyst using flowFlowJo in order to

1. produce summary tables of the names and numbers of gates described in the workspace(s),
2. execute the complete set of gatings described in the workspace, returning a comprehensive table of summary statistics for all of the populations for each of the channels,
3. obtain a set of ordered lists of FCS file paths, spillover matrices, and flowCore S4 filter objects identical with that created by the researcher using FlowJo. These objects can then be used in a more detailed event-level analysis than would be possible from simple summary statistics alone.

**Figure 1:**
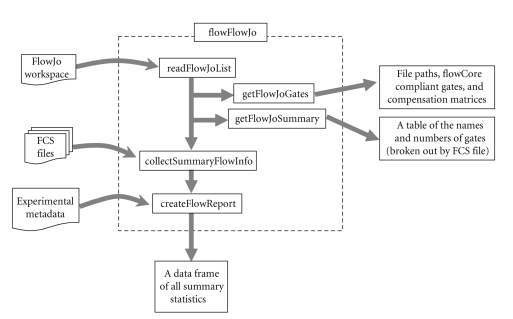
Diagram of the major methods of the flowFlowJo package and their relationship in typical use.

Figure 1 illustrates how the major components of the flowFlowJo package are related in typical data analysis sessions. The following code examples demonstrate part of such an R session using flowFlowJo to analyze a set of cytometric data. In the first line of this example the analyst reads in a FlowJo workspace from a file on his system. In the second line the analyst obtains a list of all the files and gate names referenced in the workspace to ensure that correct number and types of gates have been obtained. For brevity, the contents of this call are not shown in the demo below. In the third line the analyst “executes” the workflow detailed in the workspace via the *collectSummaryFlowInfo* command to assemble a complete set of summary statistics on all of the FCS files and all of the gates described in the workspace. In this example, the analyst also instructs the code to recover the photomultiplier tube voltage setting as recorded in each FCS file via the keywords argument. In fact, the keywords argument allows the analyst to recover any of the metadata embedded within the header section of each FCS file. The list of possible keywords and their values can be found for any FCS file with the standard flowCore call, *keyword*. In the fourth line of code, the analyst converts the complex summary object to a standard R data structure while merging it with additional metadata describing experimental details:

fjListObj <- readFlowJoList("C://Documents and Settings/TestFlowJoFile.wsp")

gateAndFileInfo <- getFlowJoSummary (fjListObj)

summaryStatsObj <- collectSummaryFlowInfo (fjListObj, keywords=c("$P1V"))

flowReport <- createFlowReport (summaryStatsObj, extraMetaDataFrame)

The analyst then works with the resulting standard R data structure to produce reports and analyses as needed. The above code provides only summary statistical information on the populations delineated in the workspace. However in some cases the analyst may wish to examine the distribution of data within a population much more carefully or gain event by event access to the cells within a population. The *getFlowJoGates* command as invoked in the first line of the example session below returns an ordered list-of-lists containing all of the file locations, file names, compensation matrices, gate names and flowCore compliant filter objects corresponding to all of the FCS files with the regular expression “Specimen.*C01” in their full pathname. As discussed above, the *getFlowJoSummary* command will return the full set of file names referenced in the in a FlowJo workspace from which the analyst may wish to choose a subset via the fileNamePatterns argument. The default for the fileNamePatterns argument returns the information for all of the FCS files referenced in the workspace. In the second and third lines below, an FCS file is loaded into memory and compensated. The fourth and subsequent lines illustrate standard flowCore operations on the associated “CD3+:Lymphocyte” filter object. The *summary* command shows the number and percent of cells recorded in the FCS file that fall within the boundaries of the CD3+: Lymphocyte gate. Finally the gate is adjusted by moving each of its forward scatter polygon coordinates 10% higher:

gateList <- getFlowJoGates(fjListObj, fileNamePatterns=c ("Specimen.*C01"))

aFlowFrame <- read.FCS (gateList$FCSFilename[[ 1]])

aFlowFrame <- flowJoCompensate (aFlowFrame, gateList$compMats[[ 1]])

aFilter <- gateList$filter[[ 1]]

aFilter

filter ‘Specimen_001_C1_C01.fcs: Lymphocytes: CD3+’

the intersection between the 2 filters

Polygonal gate ‘Specimen_001_C1_C01.fcs: Lymphocytes' with 6 vertices in dimensions FSC-A and SSC-A

Rectangular gate ‘Specimen_001_C1_C01.fcs: CD3+’ with dimensions:

Pacific Blue-A: (337.211599131617, 5996.56443562053)

PE-A: (11.0542047560856, 37903.8875296341)

summary(filter(aFlowFrame, aFilter))

Specimen_001_C1_C01.fcs:Lymphocytes: CD3++: 14342 of 99286 events (14.45%)

summary(filter(aFlowFrame, aFilter@filters [[ 1]]@boundaries [,"FSC-A"] ∗ 1.1))

Specimen_001_C1_C01.fcs:Lymphocytes:CD3++: 13043 of 99286 events (13.14%)

As can be seen, the types of operations that can be conducted at this point are virtually limitless. The *getFlowJoGates* method simply provides the user with all of the relevant components found in the FlowJo workspace as R and flowCore compliant objects in a set of commonly ordered lists.

### 2.2. File Locations, Gates/Filters, Spillover Matrices Compensation Matrices and Transformations

Prior to using the flowFlowJo package, FlowJo will have been used to manually process (compensate and gate) one or more FCS files to produce one or more FlowJo workspaces. This is a routine process for those analyzing flow cytometry data. Worth noting is that the location of the FCS files is stored in the FlowJo workspace as absolute or relative paths. Moving the FCS files to another location will cause the location of these files as extracted from the workspace to be in error and further processing steps on these files will be impossible. In anticipation of this possibility, the *readFlowJoList* method allows the user to specify an alternate path for the referenced FCS files.

 It is common practice that an assay is performed over many weeks or months, with the data from each day's run being accumulated into a single FlowJo workspace. Furthermore, it is not uncommon for the files containing the data from various runs to be given the same names. The package flowFlowJo allows for this by reading in any number of FlowJo workspaces at the same time and tracking the location of the FCS files by their full pathname.

 Some inconsistencies appear in the use of terminology in flow cytometry software and literature with respect to compensation matrices. FlowJo workspaces include sections labeled “CompensationMatrix” which are more properly referred to as “spillover” matrices. The spillover matrix elements represent the proportion of the signal emitted by each fluorescent dye that falls within the band pass windows for each of the other fluorescent dyes. The compensation matrix is the inverse of this matrix. Currently, in order to obtain similar results (e.g., mean fluorescent intensities and cell counts) between FlowJo and flowCore, it is necessary to multiply the observed signal values by the spillover matrix to the data with the usual flowCore method call (*compensate*) and then to divide all of the observed fluorescent (nonscatter) data by the maximum of the values in the spillover matrix. The flowFlowJo package implements an internal method, *flowJoCompensate*, to automatically take care of this issue when generating summary statistics. It is also worth noting that FlowJo (and flowFlowJo) allow for a different spillover matrix for each FCS file referenced within each workspace.

 Standardized interpretation of the gating coordinates can also be problematic. The information contained within the DivaSettings and TransformSettings sections of the FlowJo workspace records the user's preference for gating visualizations. This data is parsed and returned by the *readFlowJoList* method. However, all the fluorescence channel coordinates are encoded by FlowJo in their nontransformed gate coordinates. Hence there are no methods in flowFlowJo that currently utilize transformation and “DivaSettings” data, since they appear to have no impact on the obtained results. Additionally, due to code legacy, FlowJo reads the scatter gate data of FCS files in only 12 bit resolution (i.e., a maximum value of 4096). However modern flow cytometers typically record integrated signal intensities at 18 bit resolution (i.e., a maximum value of 262143). Thus the forward and side scatter gate coordinates are currently (FlowJo 7.2.5) encoded as 1/64 of their actual values for 18 bit FCS files. In these cases the *readFlowJoList* method automatically (internally) multiplies each of the scatter gating coordinates by 64 to adjust for this prior to generating flowCore filter objects.

### 2.3. Data Summary Objects

The first step in automating the analysis of manually gated data is to ensure uniformity of the naming convention across all of the samples and to confirm that all of the expected data is present. With larger data sets, problems may include (1) different names for the same cell populations, (2) missing gates, (3) missing samples, and (4) unexpected gates or samples. Such unanticipated deviations from the experimental plan can become buried in a large set of data and often compromise the downstream data analysis. A simple summary of the data is useful for identifying these anomalies. Toward this end, the *getFlowJoSummary* method returns a table showing the number and counts of different gate names associated with all of the FCS files in one or more FlowJo workspaces.

 In some cases, a data analyst may wish to proceed manually in R with the organized lists of FCS files, filters, and spillover matrices extracted by flowFlowJo. This can be accomplished with the *getFlowJoGates* method described above. However, in many cases, the analyst may be satisfied with the gating choices created in FlowJo, and may wish to simply acquire a complete set of summary statistics on all of the cell populations. The FlowFlowJo package provides methods to automatically “execute” the gating strategy provided in the workspace. It is only at this point that the flowFlowJo methods actually access the FCS files. The *collectSummaryFlowInfo* method systematically employs standard flowCore methods to create a data structure summary object with median fluorescent intensities and cell counts for each of the channels for each of the populations, as well as any requested header information from each of the FCS files.

 Each FCS file is composed of several sections in addition to the raw list-mode data. The header section of each FCS file typically contains 100 or more pieces of information about each flow run, including laser settings, photomultiplier tube voltages, run times, and other information. The *collectSummaryFlowInfo* method can be configured to collect one or more of these items from each FCS file. As a practical matter, since each FCS file may be quite large, the code only reads one FCS file into memory at a time, extracts the appropriate information, and frees its memory before moving on to the next file.

 Finally, the *createFlowReport* method can combine the summary object with additional metadata about the experiment such as sample information or treatment conditions. The resulting flow report will contain one line for each channel of each cell population of each FCS file along with any associated metadata and keywords from the header section of the FCS file.

 There are a wide variety of possible gate types within FlowJo. The current version of flowFlowJo can process range, rectangle, polygon, quadrant, and “auto” gates. Elliptical gates are not currently supported. With the advent of FlowJo version 7.5, the gate descriptions in the workspace are expected to be consistent with the Gating-ML standard [8], and we will be upgrading flowFlowJo to handle all gate types produced by FlowJo. Additional detail on the use of flowFlowJo is contained in the vignette that is available through Bioconductor (http://www.bioconductor.org/).

## 3. Applications

In the following sections we describe two applications in which we believe it is beneficial to have computational access to manually defined gates. These two applications are intended to illustrate how flowFlowJo, by allowing for computational access to manually defined gates, will make it easy to address questions and concerns about gates and the gating process. We hope that flowFlowJo will provide for an easier comparison of manual and automated gating approaches and improve our confidence in different gating procedures.

### 3.1. Supporting Reproducible, Semiautomated Flow Cytometry

In our experience, flow cytometry is commonly practiced in one of two ways. The first way occurs when a small number of samples are evaluated as part of an ongoing process of hypothesis generation and testing. The second way occurs in the clinical lab, a highly-regulated, high-throughput environment, in which there is little room for exploration or follow-up. In our view there is an important need for a third option in flow cytometry. This third way (which we call reproducible, semiautomated flow cytometry) supports the manual, exploratory analysis which is easy to do in software tools such as FlowJo or FCSExpress, but also allows for the type of modeling and analysis that has proven beneficial in the microarray arena. In addition, reproducible, semiautomated flow cytometry should have the potential to retain, and even improve upon, many of the benefits available in the highly regulated clinical environment.

 The flowFlowJo package supports a reproducible, semiautomated system in three primary ways. First, the package supports the use of FlowJo, which provides the bench researcher with a familiar tool for the visualization and exploration of flow data. Secondly, flowFlowJo moves all computations on the original data set into the R programming environment—thus allowing for automation and reproducibility of analysis statistics [9]. These summary statistics can then be readily exported into other visualization tools such as SpotFire. Third, the availability of all the data in R allows the use of a wide range of sophisticated statistical analysis tools. Data visualization and analysis often raises questions pertaining to the gating of cell populations. These questions can be readily explored because all of the postgating analyses can be automated.

### 3.2. Gating Robustness

Gating is an important and often time-consuming component of the analysis of large flow cytometry data sets. The delineation of the boundaries of cell populations is often made difficult by variable numbers, size, shape, and location of both target and nontarget cell populations. This variability may be due to debris arising from problems with sample handling or reagents, or may be due to changes in cell populations arising from disease or specific genetic differences. These problems may only become apparent in the midst of a large project, and it can be problematic to preemptively design an algorithm or model capable of handling such unforeseen problems. The difficulty of automating the pattern recognition of (potentially) distorted objects in the presence of noise is recognized in other fields [10] as well, in which the human ability to identified distorted words and characters is relied upon. While manual gating is relatively robust to unanticipated cell population distributions, it suffers from the potential for operator bias. In fact all gating methods have their drawbacks in particular cases, and tools and procedures are needed for evaluation of the results of the gating process.

 It is important to be able to assess the robustness of gating results irrespective of the method employed, and some relatively robust approaches do exist [11, 12]. In general, the results of an experiment are considered robust if they are not sensitive to small changes in the assumptions or methods used to arrive at the results. To assess gating robustness in flow cytometry, it is extremely useful to be able to work with gates in a computational framework. There are at least three intertwined aspects of gating robustness that are important to assess: gating method, gate method tuning, and gate homogeneity. For illustrative purposes, we focus on the first and only briefly comment on the other two.

 As an illustration of the assessment of gating method robustness, we examined a set of human blood samples run in a single 96-well plate. These samples originated from blood drawn from four healthy donors that were stimulated *ex-vivo* with various levels of TNF-*α* by three operators (resulting in a total of 96 samples) as part of an assay development program. The samples were stained with a variety of different antibodies, of which we only consider the antibodies for CD3/CD14 and P-p38 (the phosphorylated form of mitogen-activated protein kinase 14) as expressed by the monocytes. The antibodies to CD3 and CD14 were both conjugated to the same dye because cells staining for either of these markers can be distinguished in the SSC channel, thus allowing for the use of more channels for other markers of interest. P-p38 is intracellular and was detected by an experimental protocol in which the cells were permeabilized.

 Monocytes were gated in several ways in order to assess the robustness of results to the choice of gating method employed. For method I gates were created manually in FlowJo using a polygon gate drawn on a SSC versus log_10_(CD3/CD14) bivariate plot. The gates for method II were obtained with a robust normal fit via the *fitNorm2* method from the R package prada [13] on the cells gated with method I. The *fitNorm2* method uses a contour level for the resulting bivariate normal distribution chosen as the gate boundary [13]. Method III found the intersection of manually gated cells from method I with regions of significant curvature obtained via the *featureSignif* method in the R feature package which fit a two dimensional probability density function [14] to all of the SSC and CD3/CD14 data for each flow file. Results were then compared across the three methods for all samples. Each of these methods has one (or more) tuning parameters which can be used to make results match very closely for any individual sample between the three different methods. It is the agreement across methods for all the samples that is of importance. For method I (manual gating) the operator created a polygon gate using as many vertices placed in whatever locations were deemed appropriate. For method II and III, the various tuning parameters were selected so as to provide results close to those obtained with method I. It should be noted that the results for methods II and III are by design subsets of the results obtained by method I.

**Figure 2:**
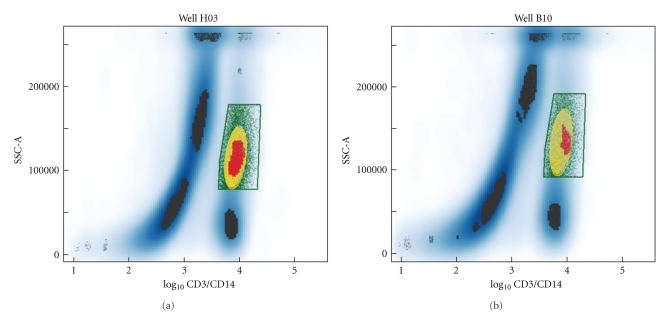
Gates for the monocyte population as produced by the three gating procedures applied to two of the 96 whole blood samples. The distribution of the cells is indicated by the blue shading with darker blue corresponding to regions containing higher numbers of cells. Regions where a probability density function fit to the data was calculated to have significant curvature are indicated in black, except where they lie within the manual gate and are colored red. Gating methods I, II, and III are shown in green, yellow, and red, respectively. The regions were colored in order of largest to smallest for visual display because the gates overlap with each other.

**Figure 3:**
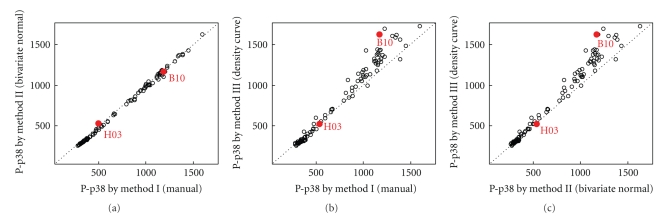
Comparison of the monocyte P-p38 mean fluorescent intensity as determined by the different gating methods for the 96 samples of whole blood. The apparent P-p38 response in any particular sample may be affected by the donor, the person running the assay, and the amount of TNF-*α* stimulation applied to the cells. The points corresponding to the two samples shown in Figure 2 are labeled in red.

Figure 2 shows the gates obtained by the three methods for two of the blood samples. These two cases bracket the range of observed agreement between gates; very good for sample H03, and poorer for sample B10. For every gate, the response of the cells in that gate as measured by their mean P-p38 levels was computed (the level of response for each sample is driven primarily by the TNF-*α* stimulation level). Comparison of response measures between the three gating methods is shown in Figure 3. It is clear that method I and method II agree very closely, while method III is moderately different from the first two methods. These simple graphs illustrate both a bias and variance between methods that should be taken into consideration in evaluating the strength of any conclusions drawn. Thus we have obtained an indication of the level of uncertainty due to gating strategy and can readily identify cases in which further investigation is warranted. Conversely, of course, we may decide one of the gating methodologies performs poorly and remove it from use for a particular application.

It is also important to look at the homogeneity of the cells within a gate. In some cases the monocyte population of the samples examined in this experiment was actually composed of two populations of cells as distinguished by different P-p38 expression. This difference was not apparent when examining the cells in the SSC versus CD3/CD14 domain. These two populations have offset centers in SSC versus CD3/CD14 space which causes a gradient in mean P-p38 expression across the manually drawn gate. Such mixed populations might be observed, for example, through use of 3 dimensional viewing tools. Alternatively one might color each point within a gate as a function of its distance in all measured parameters from the center of the gate, thereby providing a simple visual measure of cell homogeneity within a gate. Another approach is to divide the gate up into a number of subsets and compute the desired summary statistic for each subset. The variability across these subsets provides an assurance of the strength of the assumption of homogeneity within the gate. These types of visualizations and analyses are readily explored when the data is available in the R statistical programming environment.

 The P-p38 heterogeneity of some of the samples illustrates the strengths and weaknesses of the three gating methods depending on the nature of the question being asked. If the goal is to further subdivide a population, it is important to be as inclusive as possible because the subtypes in other gating parameters may not be uniformly scattered across the parent gate. If the goal is to perform a dose-response assay by measuring, for example, the phosphorylation state of an internal signaling protein, a more restricted population such as the bivariate normal gate (method II) might be more appropriate. The curvature gradient approach (method III) is particularly sensitive to the distribution of cells within a region and might be valuable in assays where detecting slight changes in the population structure is important. The gating method and tuning parameters chosen should be chosen based on the question being addressed.

 Finally, other aspects of the gating process may likewise be assessed for robustness. Automated and partially automated approaches have tuning parameters that are usually set to work well for test cases. The sensitivity of the results in these test cases can be helpful in judging how well the approach is going to work in a full study. To assess this sensitivity, an approach similar to that shown above can be used to systematically vary the tuning/controlling parameter and assess the variability in the results as a function of the controlling parameters.

## 4. Discussion and Conclusion

As the number of flow cytometry data sets grow in a study, it becomes increasingly difficult to explore “what-if” questions. It is common to uncover a behavior that can only be investigated by creating new gates or adjusting existing gates. Exploration and analysis of a data set can also reveal problems with an initial gating strategy that can be easily fixed computationally, but would be tedious to fix manually. Examples of this include the case in which manual gates are refined computationally and the case in which the robustness of gates (drawn manually or computationally) is assessed. We have also experienced cases in which a population that initially appeared to be of little importance turned out to be of substantial interest. In one case, the population had been poorly gated, and the events at the maximum possible intensity were included, but should not have been. Rather than re-gating manually, it was simple to adjust each gate computationally to exclude the boundary region.

 The flowFlowJo package provides a set of methods for extracting and organizing information from FlowJo workspaces and the FCS files to which they refer. In its most basic application, it allows the user to retrieve all of the gates and spillover matrices for all of the FCS files described within one or more FlowJo workspaces. The gates are returned as flowCore compliant filter objects, and the spillover matrices are returned as numeric matrices. Additional functionality is gained by the ability of the user to effectively run all of the compensation and gating functions described by the workspace(s) and automatically retrieve all of the relevant summary statistics into a concise data structure. These data may also be easily combined with any metadata describing the nature or source of each sample and any experimental conditions to which they were subjected.

 There has been limited involvement by the bioinformatics, statistical, and machine learning communities in the problems of flow cytometry [15]. Programmatic access to both raw data and gates in flow cytometry allows us to ask many questions about flow cytometry data that traditionally were tedious or effectively impossible. The ability to assess gate choice assumptions is expected to lead to better assessments of the quality of our methods. In some cases, more sophisticated approaches such as mixture modeling may be called for when seemingly uniform cell populations actually include two or more cell types. This is especially important when examining cell populations for which a subset of the cells with no known defining antibodies respond differently to stimuli than the rest of the cells in the population.

 At the present time, flowFlowJo is known to work with FlowJo version 7.2.5 running on the Windows operating system. We expect FlowJo to continue to evolve and we intend to maintain flowFlowJo in such a way that it can handle the current FlowJo workspaces. A major change in the FlowJo workspace structure will be the transition to FlowJo 7.5 when the use of the Gating-ML standard is expected to replace the current XML format. The flowFlowJo package and supporting vignette and documentation is available from the Bioconductor web site (http://www.bioconductor.org/).
